# Pathogenic and Nonpathogenic Antibody Responses in Allergic Diseases

**DOI:** 10.1002/eji.202249978

**Published:** 2024-12-29

**Authors:** Marc Ehlers, Friederike Jönsson

**Affiliations:** ^1^ Laboratories of Immunology and Antibody Glycan Analysis Institute of Nutritional Medicine University of Lübeck and University Medical Center of Schleswig‐Holstein Lübeck Germany; ^2^ Airway Research Center North German Center for Lung Research (DZL) University of Lübeck Lübeck Germany; ^3^ Institut Pasteur Université de Paris Cité, Unit of Antibodies in Therapy and Pathology Paris France; ^4^ CNRS Paris France

**Keywords:** allergy, antibody glycosylation, IgE and IgG, pathogenic versus nonpathogenic antibody responses, tolerance versus immunopathology

## Abstract

Allergen‐specific antibodies, particularly of the IgE class, are a hallmark of many allergic diseases. Yet paradoxically, (1) a proportion of healthy individuals possess allergen‐specific IgE without clinical signs of allergy; (2) some, but not all, allergic individuals develop a more severe disease over time or fail to respond to allergen‐specific immunotherapy; and (3) allergen‐specific IgG antibodies can inhibit IgE‐mediated responses but they can also induce allergic reactions. In this review, we discuss the occurrence of and transition between nonpathogenic and pathogenic allergen‐specific antibody responses in the light of a two‐stage model. We recapitulate different factors and scenarios that may induce different inflammatory conditions and qualitatively distinct allergen‐specific T‐ and B‐cell responses, influencing IgE origins and affinities, IgE/IgG(4) ratios, IgG effector functions, antibody glycosylation patterns, Fc and glycan‐binding receptor expression and involvement, and ultimately their propensity to elicit allergic responses. Differences in these antibody characteristics may determine the onset of symptomatic allergy and the severity or remission of the disease.

## Graphical abstract

Allergen‐specific antibodies can be detected in a large proportion of individuals. Still, only a fraction suffer from clinical signs of allergic disease, and of these, some, but not all, will develop more severe forms of the disease over time or respond to allergen‐specific immunotherapy. Here, we review the concept of nonpathogenic versus pathogenic antibody responses in allergy and discuss the impact of environmental factors and inflammatory conditions on shaping antibody responses in allergic individuals.

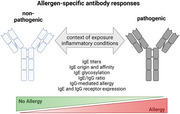

**Abbreviations**
Ab: antibody
AIT: allergen‐specific immunotherapy
galectins: beta‐galactoside‐binding lectins/S‐type lectins
GC: germinal center
IC: immune complex
Ig: immunoglobulin
sial: sialylated
siglecs: sialic‐acid‐binding immunoglobulin‐like lectins

## 1 Introduction

Many allergic affections are characterized by the development of pathogenic allergen‐specific T and B (T/B) cell and antibody (Ab) responses (type I‐IV allergies [1]). The initial exposure to an allergen that induces allergen‐specific Abs is called the sensitization phase. Allergen‐specific IgE Abs, the inducers of the most common type I allergic diseases [1], then bind to the high‐affinity FcεRI on mast cells and basophils, even in the absence of allergen. At the next allergen contact, the allergen can then aggregate the IgE/FcεRI complex and induce the release of inflammatory mediators within seconds or minutes, the so‐called effector phase. In addition, IgG can induce allergic responses by binding to cell‐bound allergens (type II diseases [1]) or by forming soluble IgG immune complexes (ICs; type III diseases [1]) in the presence of higher antigen concentrations and subsequently activating complement and/or cross‐linking classical activating FcγRs on myeloid cells [2, 3, 4, 5].

However, there is accumulating evidence that an unquantified proportion of individuals can possess allergen‐specific IgE or IgG (i.e., be sensitized) without developing disease upon the next allergen challenge and that some, but not all, allergic individuals develop more severe disease over time or fail to respond to allergen‐specific immunotherapy (AIT) [4, 6, 7]. Accordingly, one modifying possibility is that in addition to their activating role, the presence of allergen‐specific IgG Abs can also reduce IgE/FcεRI‐mediated cell activation by competing with IgE for allergen epitopes (allergen masking) or by co‐engaging the allergen/IgE/FcεRI complex with the inhibitory IgG receptor FcγRIIB [8, 9].

Therefore, it has been proposed and shown that both the sensitization phase, as well as the effector phase of each individual, can differ in their allergen‐specific T/B cell and Ab responses, that is, nonpathogenic or low or high pathogenic (Ab) immune responses, which may abolish or induce an allergic effector phase.

## 2 A Two‐Stage Model for the Induction of Nonpathogenic Versus Pathogenic Allergen‐Specific Antibodies

**FIGURE 1:**
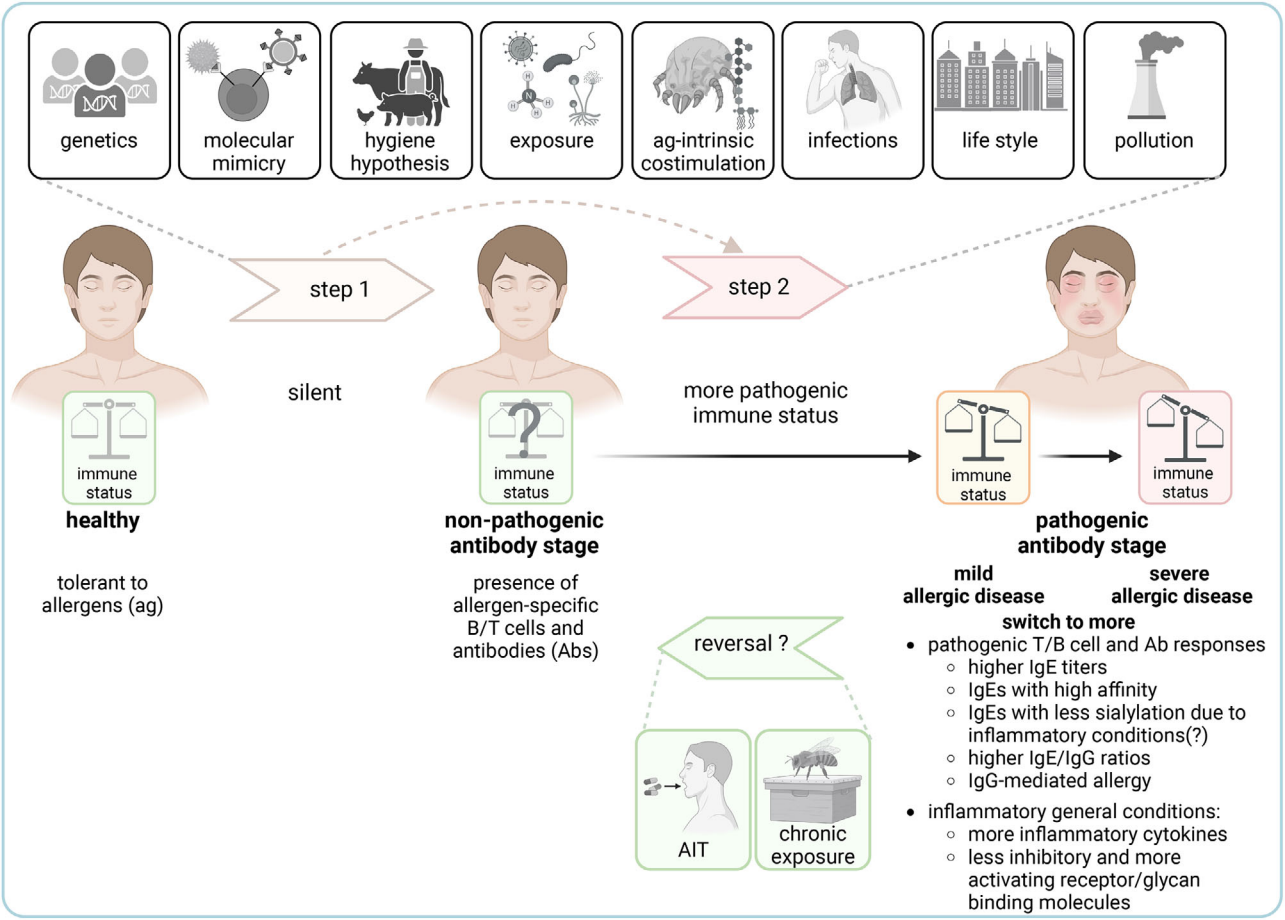
Two‐stage model for the induction of nonpathogenic versus pathogenic allergen‐specific antibodies. See the text for an explanation.

Different factors and scenarios may influence the general inflammatory immune conditions and Ab titers, origins, affinities, longevity, IgE/IgG ratios, IgG subclasses, and IgE and IgG subclass Fc glycosylation patterns, which may influence the Ab composition and capacity to abolish or activate allergic responses, resulting in a nonpathogenic or pathogenic T/B cell and Ab stage (Figure 1).

In addition, further observations now suggest that these two stages are likely to have dynamic transitions. Some of the sensitized healthy individuals with initially nonpathogenic Abs may develop pathogenic Abs and allergies over time; accordingly, some allergic patients may have been sensitized years before developing allergic symptoms; and patients receiving AIT may revert to a non‐pathogenic stage (Figure 1). In this review, we summarise data and arguments in favor of such a dynamic two‐stage model and discuss its limitations in the context of Ab‐driven allergic diseases (type I‐III allergies [1]).

Lessons from other Ab‐dependent diseases may explain such a dynamic two‐stage model. It is increasingly accepted that inflammatory autoimmune diseases can also show a nonpathogenic and a pathogenic stage with dynamic transitions [10, 11]. In the first step, tolerance mechanisms fail, and self‐reactive T/B cell responses lead to detectable (IgG) autoantibodies. However, these autoantibodies are generally not associated with clinical signs. In the second step, this specific immune response shifts to an inflammatory T/B cell and Ab response and clinical symptoms in a (minority) proportion of individuals. Step two may occur concurrently with step one or years later.

Ab‐driven allergic diseases could likewise develop in a two‐step process (Figure 1). Interindividual factors and scenarios are likely to determine if and when an individual (i) loses tolerance to allergens and induces innocuous allergen‐specific T/B cell and Ab responses (stage 1) and (ii) shifts from harmless to pathogenic allergen‐specific T/B cell and Ab responses and overt allergic disease (stage 2). Nonpathogenic Ab responses could evolve over time—for example, driven by more inflammatory conditions—into more pathogenic Ab responses (stage 2). Alternatively, a healthy individual could develop a stage 2 allergic immune response without a significant stage 1 response.

Intrinsic factors, as well as the context, in which an individual is exposed to an allergen, determine the induction and progression of allergic disease; they can either induce the development of clinically silent immune responses (stage 1), the evolution from stage 1 to stage 2, or the direct transition to allergic immune responses (stage 2; Figure 1). These factors include:

- Genetic predisposition, polymorphisms, and epigenetic modifications [12].
- Exogenous molecules from microbes with structural similarities to potential allergens (molecular mimicry) [13].
- An overly “aseptic” environment, especially in early life, contextualized in the hygiene hypothesis, resulting in low immune occupancy, which is thought to favor inappropriate immune activation [14, 15, 16].
- Exposure to a lower diversity of microorganisms under urban conditions—including fewer anti‐inflammatory species—resulting in a more proinflammatory immune status and more allergic diseases than under rural conditions [15, 16].
- Allergen‐intrinsic co‐stimulation, such as that reported for the major house dust mite allergen, Derp2, can co‐activate Toll‐like receptor (TLR) 4 [17].
- Allergen exposure in the presence of an ongoing infection or inflammation, allowing allergen‐specific B cells to receive bystander help from immune activation and irrelevant T cells [18].
- Environmental factors such as air pollution can act as inflammatory co‐stimulants [19] or modify “harmless” allergens to make them more immunogenic [20, 21] through nitration and oligomerization.
- Other environmental (lifestyle) factors, such as obesity, diet, and stress, can increase inflammatory conditions [15].

AIT or chronic exposure to allergens (such as bee venom in beekeepers), if successful, can restore a tolerogenic stage, in which individuals do not develop symptoms upon re‐exposure to allergens despite the continued presence of allergen‐specific IgE Abs (Figure 1). This is due, at least in part, to the induction of IgG(4) Abs. A shift in the IgE/IgG(4) ratio is only one hallmark of AIT, which profoundly remodels the immune landscape and is reviewed elsewhere [22, 23].

Future studies need to clarify how and to what extent these different factors and conditions contribute to the silent breakdown of tolerance and the induction of more general inflammatory conditions and/or pathogenic T/B cell and Ab responses in the context of type I–III allergic diseases. Several characteristics of nonpathogenic versus pathogenic allergen‐specific Ab responses have been shown and suggested to play a role in the context of IgE‐ or IgG‐mediated allergic reactions.

## 3 Nonpathogenic Versus Pathogenic IgE Antibodies in Allergic Reactions

**FIGURE 2:**
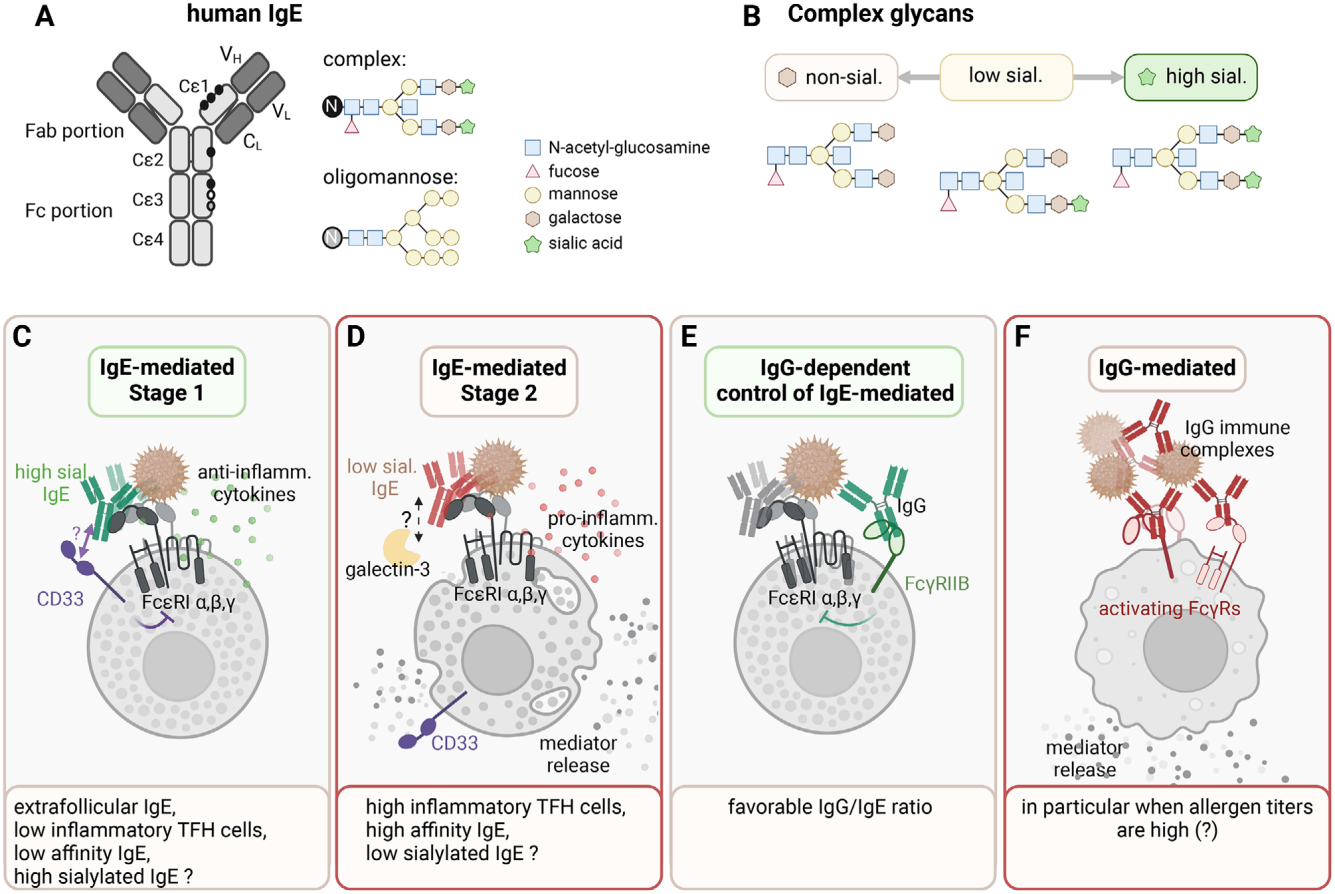
Nonpathogenic versus pathogenic allergen‐specific antibody responses. (A) Human IgE possesses seven glycosylation sites: five (black circles) carry a complex glycan structure, one (grey) an oligomannose glycan, and the white site is unoccupied [40, 41, 50]. (B) Examples of complex glycans with no, low, and high sialylation (sial.). (C) Conditions of nonactivation of mast cells and basophils (stage 1) including the hypothesized inhibitory effect of highly sialylated IgE through CD33 (see text). (D) Conditions of mast cell and basophil activation (stage 2) including the hypothesized activation by low sialylated IgE and galectin‐3 (see text). (E) IgG‐dependent control of IgE‐mediated mast cell and basophil activation by crosslinking FcγRIIB (see text). (F) IgG‐mediated immune cell activation by activating FcγRs (see text).

IgE binds with high affinity to FcεRIα [24, 25] on mast cells and basophils, which is complexed with FcεRIβ and FcεRIγ. Upon aggregation by allergens, this complex can trigger the release of inflammatory mediators (Figure 2). Multimeric IgE ICs can also bind to the low‐affinity IgE receptor FcεRII (CD23). The interaction of IgE with CD23 regulates several effector functions on immune cells [26]. On B cells, membrane‐bound CD23 downregulates IgE production through a negative feedback loop upon interaction with IgE [26]. In the circulation, soluble (shedded) CD23 enhances IgE production by blocking its interaction with membrane‐bound CD23. Interestingly, IgE binds to FcεRIα and CD23 with different conformations [27]. Activation of these pathways can be enhanced or inhibited by inflammatory or noninflammatory cytokines, for example, by regulating the expression levels of these receptors and other molecules [28].

The two‐stage model also assumes that there are qualitatively different allergen‐specific IgE Ab responses.

Extrafollicular T/B cell responses have been associated with the induction of un‐/low‐mutated IgE with low affinity and no allergic or even anti‐allergic potential, whereas germinal center (GC)‐dependent B cell responses leading to highly mutated IgE with high affinity have been linked to the induction of allergic disease [29, 30, 31] (Figure 2). The latter B cell responses are instructed by T follicular helper (T_FH_) cells through cytokine release and contact‐dependent signaling [32] (Figure 2); a specific T_FH_ subpopulation, T_FH13_, has recently been linked to the induction of anaphylactic IgE Abs [33, 34].

In addition, the biological activity of IgE Abs is influenced by their type of glycosylation, which can affect their structure, as well as their interaction with IgE receptors and additional membrane‐bound and soluble carbohydrate‐binding proteins (e.g., galectins (beta‐galactoside‐binding lectins/S‐type lectins), siglecs (sialic acid‐binding immunoglobulin‐like lectins), and C‐type lectin receptors) [35, 36, 37, 38, 39] (Figure 2). Human IgE has seven and mouse IgE has nine conserved *N‐*glycosylation sites in the constant region of each heavy chain (Figure 2A). Most of these are of the complex type and potentially galactosylated and terminally sialylated (sial) [40, 41] (Figure 2B).

Interestingly, recent murine and human studies have shown that extrafollicular plasma cell (PC) responses induce highly galactosylated and sialylated IgG Abs, whereas GC‐derived B cell responses induce lower galactosylated and sialylated IgG Abs [42, 43, 44]. Whether this is also the case for IgE responses remains to be investigated.

Furthermore, different inflammatory conditions can further modify the galactosylation and sialylation levels of GC‐derived IgG Abs. In the context of inflammatory (auto)immune diseases, it has been shown that the induction of highly inflammatory cells, for example, IL‐17‐producing T helper cells and T_FH17_ cells, or a reduction of regulatory T cells induces more inflammatory IgG responses that trigger or exacerbate the disease [10, 11, 45, 46]. More precisely, inflammatory GC responses in the presence of T_FH17_ cells and reduced T follicular regulatory cells favor the secretion of IgG with low levels of galactosylation and sialylation, which are linked to inflammatory (auto)immune conditions [43].

Different natural or AIT‐induced inflammatory states of the specific T/B cell response, as described for IgG [43, 47, 48], may lead to differently glycosylated GC‐derived IgE with divergent potential to induce allergic responses, which remains to be investigated (Figure 2C,D).

In the context of allergy, the induction of T_H17_ cells has also been linked to more severe responses, particularly in asthma [49]. It is therefore tempting to hypothesize that the induction of inflammatory GC responses may also favor the induction of IgE Abs with low levels of galactosylation and sialylation.

By comparing the effects of sialylated and enzymatically de‐sialylated monoclonal IgE Abs, Shade et al. [50] suggested that highly sialylated IgE has a greater potential to induce allergic reactions than less sialylated IgE. In contrast, Dühring et al. reported that low sialylated IgE bares a higher mast cell and basophil activation potential than high sialylated IgE [51]. The second finding would be consistent with findings that inhibitory effects of protein sialylation evolved long before the adaptive immune system and that sialylation of IgG, IgA, and IgM attenuates their inflammatory potential [43, 48, 52, 53, 54]. The second statement would also be more consistent with the idea that extrafollicular B cell responses generate highly galactosylated and sialylated (IgE) Abs and GC‐derived B cell responses generate less galactosylated and sialylated (IgE) Abs and that inflammatory conditions during the GC response may further downregulate (IgE) Ab galactosylation and sialylation.

However, in the absence of conclusive in vivo confirmation, further studies are needed to clarify the role of different glycosylated IgE Abs. Interaction with glycan‐binding molecules will also play an important role.

CD33 (siglec‐3) is a tyrosine‐based inhibitory motif transmembrane receptor that can interact with sialic acids. Using nanoparticles with allergens and sialic acid analogs to cross‐link the IgE/FcεRI complex with CD33 can reduce mast cell activation [35]; however, there are no studies to date that have investigated whether the degree of IgE sialylation affects the ability of CD33 or other Siglecs to reduce mast cell activation, which remains to be done (Figure 2C).

In inflammatory conditions, increased expression of the IgE binding protein (galectin‐3) in its soluble form has been linked to enhanced IgE‐mediated responses [55]. In addition, galectin‐3 shows a stronger interaction with less sialylated IgE Abs [38, 56, 57]. However, evidence that galectin‐3 can specifically enhance the allergic potential of less sialylated IgE Abs in vivo is lacking and controversial [58, 59], and further studies are needed to clarify its role (Figure 2D).

Another lectin, galectin‐9, has been reported to interact with the IgE Fab part, probably in a glycosylation‐dependent manner, and to inhibit the interaction with allergen ligands [28].

In addition, recent studies suggest that differentially galactosylated and sialylated IgG Abs may play distinct activating roles on different effector cells and complement pathways, for example, with strong activating “inflammatory” effects of non‐galactosylated IgG Abs on neutrophils and complement via the mannose‐binding lectin pathway, but more activating potential of galactosylated and sialylated IgG Abs on NK cells and via C1q [44]. Whether differentially galactosylated and sialylated IgE responses may also have distinct activating effector mechanisms remains to be investigated. Furthermore, the induction and role of IgE fucosylation and bisection are poorly understood.

Taken together, the induction and function of low versus high‐affinity IgE as well as differentially glycosylated IgE appear to be important factors in the initiation (or inhibition) of allergic responses. Future studies are needed to investigate how differentially glycosylated IgE Abs develop and mediate their functions and how the induction and function of IgE glycosylation can be exploited for diagnostic and therapeutic approaches.

## 4 Nonpathogenic Versus Pathogenic IgG Antibodies in Allergic Reactions

In both humans and mice, IgE production is preceded or accompanied by the production of IgG, which can dampen IgE/FcεRI complex‐mediated allergic reactions through allergen masking and/or cross‐linking with the inhibitory IgG receptor FcγRIIB (Figure 2E) [8, 9, 24, 48, 60, 61, 62, 63].

There is increasing evidence that IgE‐producing B cells develop primarily from pre‐existing IgG+ B cells, rather than by direct class switching from naïve IgM+ B cells [30, 31, 64]. Recently, two papers have suggested that in humans, a subpopulation of “type 2–marked” IgG+ memory B cells may be precursors of high‐affinity pathogenic IgE‐producing PCs [65, 66]. Accordingly, these IgG+ memory B cells transcribe not only high levels of CD23 and IL‐4Rα but also germline IgE (*IGHE*; without producing fully rearranged IgE B cell receptors). Allergen‐specific B cells in children with peanut allergy and patients with allergic rhinitis were enriched for these “type 2–marked” IgG+ memory B cells [65, 66]. Interestingly, these cells were not only IgG1+ memory B cells but were also enriched for IgG4+ memory B cells, suggesting a complex interaction between the three Ab isotypes [65]. It remains to be investigated whether human IgG4, which increases with repeated (high) allergen exposure or AIT and is mainly associated with the inhibition of IgE‐mediated allergic reactions, also develops from “type‐2 marked” IgG1+ memory B cells or independently. Importantly, the IgE/IgG ratio is an essential factor in the regulation of IgE responses, and an increased IgE or IgG4 switching may contribute to a shift from a nonpathogenic stage to a pathogenic stage or vice versa.

Highly inflammatory immune conditions have been shown to promote the expression of activating IgG receptors and reduce the expression of the inhibitory FcγRIIB on immune cells [67, 68, 70]. This can shift the tightly regulated balance in favor of cell activation and further reduces the ability of IgG to dampen IgE‐mediated allergic responses (Figure 1). IgA may also exert inhibitory functions on IgE‐mediated allergic responses [70] but has been less studied in this context.

Opposing this regulatory function of IgG, accumulating evidence suggests that IgG is also capable of inducing allergic responses in humans through cellular cytotoxicity (type II reactions [1]) or in the form of soluble ICs (type III reactions [1]). The latter reaction has mostly been reported in the presence of high allergen concentrations that enable the efficient formation of ICs and activation of FcγRs, for example following intravenous infusion of a drug in humans [3, 4, 5] (Figure 2F).

In mice, the most prominent example of such allergic reactions is anaphylaxis, for which IgE‐ and IgG‐dependent pathways have been identified [62, 71]. Murine IgG‐anaphylaxis can be triggered either by transfer of specific IgG followed by an i.v. injection of its cognate antigen (passive systemic anaphylaxis [PSA]) or by immunization with an antigen in the presence of an adjuvant, followed by an i.v. challenge with the antigen (active systemic anaphylaxis) [48, 72]. Notably, IgG‐anaphylaxis occurs in the absence of IgE, FcεRI, mast cells, or histamine [71, 73, 74]. FcγRIIB inhibits mouse IgG‐PSA unless induced by IgG2a [75]. IgG Fc sialylation reduces the affinity of mouse IgG for activating FcγRs [53] and limits the severity of mouse IgG(1)‐PSA [69]. Notably, at least in mice, an IgG‐mediated allergic response has also been induced by small amounts of antigen [76].

IgG‐PSA involves the activation of most myeloid (FcγR‐bearing) cells. Their relative contribution to the reaction remains controversial and appears to be model‐dependent (reviewed in [77]). Once activated, these cells release platelet‐activating factor (PAF) and histamine, which are also responsible for the signs associated with IgG‐anaphylaxis in mice [75].

Transgenic mouse models enabled to demonstrate that human activating FcγRs are likewise able to induce IgG‐anaphylaxis, with human FcγRIIA playing a dominant role [78, 79]. Transgenic expression of human FcγRIIA in mice reproduces the human expression pattern, including its expression on all myeloid cells and platelets. Human FcγRIIA‐expressing platelets are activated in mice by IgG ICs and release the vasoactive mediator serotonin, which determines the severity of IgG‐anaphylaxis [80, 81, 82].

Paralleling the mouse studies, IgG receptor internalization, myeloid cell activation, elevated levels of circulating PAF (all of which correlated with anaphylaxis severity), and serotonin release by platelets following IgG‐mediated activation have also been reported in humans [4, 5, 80, 83].

Taken together, allergen‐specific IgG can exert inhibitory as well as activating functions and may trigger anaphylaxis, at least at high allergen concentrations. Further studies are needed to investigate the role of IgG subclasses and their glycosylation patterns as well as the consequences of their interaction with inhibitory and activating FcγRs on platelets, B cells, and other myeloid cells in humans.

## 5 Therapy and Tolerance Induction

Various therapeutic strategies aim to reduce allergic responses. These include depletion of IgE from the blood (plasmapheresis) [84], inhibition of its interaction with FcεRIα by monoclonal blocking Abs (e.g., omalizumab) [85], and blocking of cytokines (IL‐4, IL‐5, IL‐13) or their receptors to reduce IgE generation [86]. It may be interesting to investigate whether blocking the effector functions of, for example, T_FH17_ cells influences IgE glycosylation and thus the severity of IgE‐mediated allergic diseases.

In addition, AIT can profoundly modify the immune system and restore a tolerogenic state in patients [22, 23] (Figure 1). Remarkably, IgE titers remain stable for some time during AIT, declining only slowly over months to years. Instead, IgG Abs are induced that compete for antigen binding or inhibit IgE effector function through FcγRIIB [8, 9, 24, 48, 61, 62].

In particular, the induction of IgG4 and possibly IgG2, both of which have rather poor Fc effector functions compared with IgG1 and IgG3, seems to correlate positively with AIT success [48, 87], although a high IgG(4)/IgE ratio alone is not sufficient to predict AIT success [88]. Of interest is whether an AIT alters the existing immune response or primarily induces a new parallel immune response, and whether different AITs with different compositions and adjuvants distinctly affect the quality, that is, the glycosylation of IgE and IgG, and the composition of IgG and perhaps IgA subclasses.

## 6 Limitations of the Two‐Stage Model

Although increasing evidence supports the idea of a two‐stage model to explain the transition from nonpathogenic to pathogenic T/B and Ab responses in inflammatory autoimmune diseases [10, 11], such studies in the context of allergy are still rare. Further research is needed to determine how silent stage one and allergic stage two differ in their T/B cell and Ab responses. Co‐analysis of the inflammatory immune status of each individual, together with their IgE and IgG subclass Ab titers, affinities, Fc glycosylation patterns and ratios as well as the expression levels of activating and inhibitory receptors/molecules, may help to characterize the two different stages with dynamic transitions and establish personalized medicine in the future.

## 7 Conclusion

Several factors such as genetic predisposition, environment, allergen characteristics, and the context of exposure can influence the development and function of nonpathogenic versus pathogenic allergen‐specific Ab responses. These factors influence cytokine expression, GC responses, IgE and IgG subclass titers, affinities, glycosylation, IgE/IgG(4) ratios, and IgG‐mediated responses via classical FcγRs, as well as general inflammatory conditions that determine the expression levels of activating and inhibitory Fc and glycan‐binding receptors/molecules. A better understanding of the factors that induce the shift from innocuous to pathogenic allergen‐specific Abs and immune states, is required to identify suitable biomarkers and improve the management of Ab‐mediated allergic diseases.

## Author Contributions

Marc Ehlers and Friederike Jönsson conceived, drafted, revised, and approved the manuscript.

## Conflicts of Interest

The authors declare no conflicts of interest.

### Peer Review

The peer review history for this article is available at https://publons.com/publon/10.1002/eji.202249978.

## Data Availability

The review manuscript does not contain shared data.
